# Nanoscale symmetry protection of the reciprocal acoustoelectric effect

**DOI:** 10.1038/s41598-026-38987-6

**Published:** 2026-02-06

**Authors:** Sandeep Vijayan, Stephan Suffit, Scott E. Cooper, Yejun Feng

**Affiliations:** Okinawa Institute of Science and Technology Graduate University, Onna, Okinawa 904-0495 Japan

**Keywords:** Surface acoustic wave; Microfabrication and device engineering; Acousto-electric effect; Non-reciprocity; Stroh's equation of motion;Symmetry, Materials science, Physics

## Abstract

Neumann’s principle states that all physical properties of a material are bound by its symmetry. While bulk crystals conform to well-defined point and space groups, phenomena at a substrate’s surface could have less apparent symmetry origins. Here we experimentally explore both reciprocal and non-reciprocal types of acoustoelectric effects driven by surface acoustic waves (SAW). The non-reciprocal acoustoelectric voltage is connected to the natural single-phase unidirectional transducer from device engineering. On the other hand, reciprocal acoustoelectric effect exists in certain SAW configurations that are of different symmetry origins. Half of the configurations have a valid reciprocity-preserving symmetry element of either a mirror plane or a two-fold rotational axis that is perpendicular to the substrate surface. The other half of the configurations do not possess a reciprocity-preserving symmetry element of the half-space but relate to the first scenario through an interchange of SAW propagation and the surface normal directions. Here, it is the symmetric construction in the equation of motion, which holds at nanoscale distances, to be responsible for the correspondence between two scenarios and extends the cases of reciprocal SAW states. This correspondence traces its physical origin to the SAW composition of compression and shear waves along two orthogonal directions respectively.

**Supplementary Information:**

The online version contains supplementary material available at 10.1038/s41598-026-38987-6.

## Introduction

Surface acoustic wave states^1,2^ have been widely utilized to explore many emergent frontiers of physical science, such as even-denominator fractional quantum Hall states^3^, quantum information manipulation^4^, single electron circuitry^5^, Dirac-cone electronics^6^, the creation and transportation of solitonic skyrmions^7^, and microfluids^8^. Propagating on the surface of a substrate but extending no deeper than one wavelength into the bulk^1,2^, acoustic waves offer a linear dispersion like that of electro-magnetic waves but with a much smaller slope that is determined by the speed of sound (in solids) instead of the speed of light. In recent decades, there has been a fast-growing effort to utilize acoustoelectric (AE) effect^9,10^ to probe spintronic topics^11–14^. Among all studies, there exist many accounts of asymmetric or non-reciprocal acoustoelectric behavior, which demonstrate different amplitudes when they are driven by oppositely traveling SAW states^11–16^. Most of these studies involve magnetic materials, so the non-reciprocal property is often attributed to ferromagnetic sample films that possess a broken time reversal symmetry^11–14^, or the metal-insulator transition that is concurrent with the paramagnetic-ferromagnetic transition^15^. In some cases, when two interdigital transducers are used, the asymmetry can be attributed to inequivalent transduction efficiencies of the interdigital transducers (IDT)^16^. As research efforts are pushing towards measurements’ sensitivity limit, a complete understanding of the non-reciprocal AE effect becomes necessary and imperative.

In the literature of SAW device engineering, asymmetric phenomena have been reported in one special type of devices named natural single-phase unidirectional transducer (NSPUDT) even though electrodes are symmetrically placed^2,17–21^. This phenomenon was attributed to the influence from the mass of IDT electrodes on the reflectivity coefficient ${r}^{(\pm )}={R}_{e}^{\left(\pm \right)}+{R}_{m}^{(\pm )}{h}_{\mathrm{metal}}/\lambda$ of SAW states, with ${R}_{e}^{(\pm )}$ and ${R}_{m}^{(\pm )}$ representing the electrical and mechanical effects respectively, $(\pm )$ of two opposite propagation directions, ${h}_{\mathrm{metal}}$ the height of metal electrodes, and $\lambda$ the SAW wavelength^2^. ${R}_{e}^{\left(\pm \right)}$ are regarded as always symmetric, while ${R}_{m}^{(\pm )}$can be asymmetric. Based on the perspective of reflection center position, Ref.^18^ evaluated certain high symmetry directions among 12 monaxial point groups (out of a total of 13 except the type *1*) but did not clarify the key symmetry reason of NSPUDT’s existence. Using numerical coupling-of-modes simulations that also include the reflection effect, Ref.^19^ explored conditions of pure standing wave state and was extended to address the NSPUDT effect^21^. Nevertheless, there remains a lack of clear symmetry logic amid misidentified symmetry elements. Overall, the general NSPUDT effect was considered uncommon in the later literature^2^ and was believed not affecting SAW propagation along high symmetry directions^2^. The unclear situation caused unnecessary discussions about the three-fold symmetry in LiNbO_3_^20^ and assigning the mirror symmetry to the substrate surface plane^19–21^.

Here working with non-magnetic conductors, we have experimentally searched for and separated the reciprocal and non-reciprocal AE effects, with the latter attributed to NSPUDT-type of SAW states. While theoretical SAW states constructed from eigenstate solutions of Stroh’s equation of motion would always satisfy the time reversal symmetry, in real devices, multiple reflections during the creation of SAW states inside the IDT can cause the non-reciprocal SAW states. The reciprocal AE effect is limited to configurations of two scenarios. In LiNbO_3_/LiTaO_3_ based devices, the first scenario has a SAW state propagating perpendicular to a mirror plane of the half-space, which can be extended to all point groups with an alternative symmetry operation of a two-fold rotation about the substrate surface normal; here the global symmetry operation directly connects two oppositely propagating SAW states. Configurations of the second scenario have a one-to-one correspondence to those of the first scenario, but with directions of the surface normal $\widehat{{\boldsymbol{n}}}$ and propagation $\widehat{{\boldsymbol{m}}}$ interchanged; all other configurations lead to non-reciprocal SAW states and AE effect. Despite a lack of global symmetry, the second scenario has its correspondence to the first scenario sustained by the symmetric construction of Stroh’s equation of motion over the nanometer scale, which can be regarded as a hidden protection of reciprocal SAW states. Our experimental results and identified cause of strain tensor symmetry are related to pure standing wave states in real devices, an important topic in many experimental studies of acoustoelectric, spintronic, and quantum transport nature.

## Results

### High-sensitivity measurement of the acoustoelectric effect

**Fig. 1:**
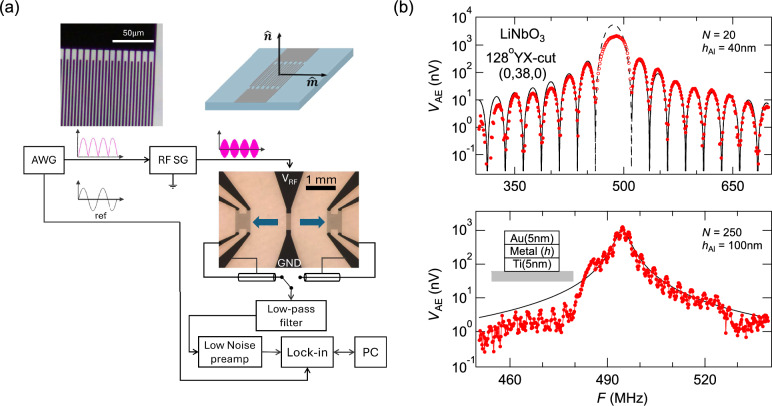
AC lock-in measurement of the acoustoelectric effect. (**a**) A schematic of the electrical circuitry (Methods). A representative device image is presented together with a zoomed-in view of IDT. Two SAWs propagate along opposite directions as marked by blue arrows. Two metal film pads are placed at equal distances from the IDT as probes of the AE voltage (Methods). The schematics of device geometry is also included, which is defined by the surface normal $\widehat{{\boldsymbol{n}}}$ and SAW propagation direction $\widehat{{\boldsymbol{m}}}$. In general, SAW simultaneously propagate along both $\widehat{{\boldsymbol{m}}}$ and $-\widehat{{\boldsymbol{m}}}$. (**b**) Acoustoelectric voltage measured at one metallic pad as a function of the input radiofrequency, for two devices at low and high limits of IDT pair number *N*. The black curves are fits to the data, using a Sinc-squared function for the *N* =20 device, and a Lorentzian function for the *N* =250 device. The fitting of the Sinc-squared form is carried out using the sidebands (solid points), as the main spectral peak (open circles) suffers a suppression from IDT mass loading.

Many past studies of the AE effect have used DC techniques that can be highly susceptible to the environment condition^11,12^. Here we detect the AE effect with an AC lock-in technique (Methods) (Fig. 1). The downshift of the measurement frequency by seven decades avoids many radio-frequency effects associated with the SAW device, such as electromagnetic waves and piezoelectricity. In a device with a low number of IDT pairs *N* (~20), we clearly observe an AE voltage profile over four decades of dynamic range (Fig. 1b) which follows a Sinc-squared functional form of a surface acoustic wave^1,2^. Thus the AE effect reflects the mechanical SAW state with a high fidelity, and the AC lock-in technique provides the high sensitivity.

The low number *N *of IDT pairs ensures that the generated SAW experiences limited internal reflections at IDT fingers before it escapes outside the IDT, as the negligence of reflection is implicitly assumed in the derivation of Sinc-square form in Refs.^1,2^. When the pair number *N* of the IDT is increased, the spectral shape evolves into a Lorentzian form (Fig. 1b)^22^. Here, even small internal reflection at each metallic finger of the IDT can have an accumulated effect within a large array. Eventually the internal reflection causes the coherence length of SAW to become shorter than the length of IDT, leading to a Lorentzian spectral form^22^. A Lorentzian fitting of the data in Fig. 1b gives a full width at half maximum of $3.04\pm 0.08$ MHz, that is clearly larger than the expected 3dB width (~$1.78$ MHz) of a $N=250$ IDT device with a Sinc-squared form^1^. The whole length of IDT remains noticeable to the SAW states, as there exist fast oscillations superposed on the overall Lorentzian form with a period about $1.85\pm 0.10$ MHz, equivalent to an estimated $N=267\pm 15$ that is consistent with the design parameter.

### Non-reciprocal AE effect and SAW states

We now probe the symmetry of the AE effect with SAW states propagating along opposite directions. Two oppositely traveling SAW states can be regarded as reciprocal to each other if they can be related by the time reversal symmetry operation. Studies in the literature often used a configuration of two in-line but distanced IDTs with one patch of metallic film in between, so each IDT can drive SAW to the same AE probe^11,15^. Here we detect the AE effect on two sides of a single IDT (Fig. 1a)^17^, using two identical patches of metallic films at equal distance away from the IDT (Methods). This configuration ensures that the oppositely propagating SAWs are created under the same transduction process.

**Fig. 2:**
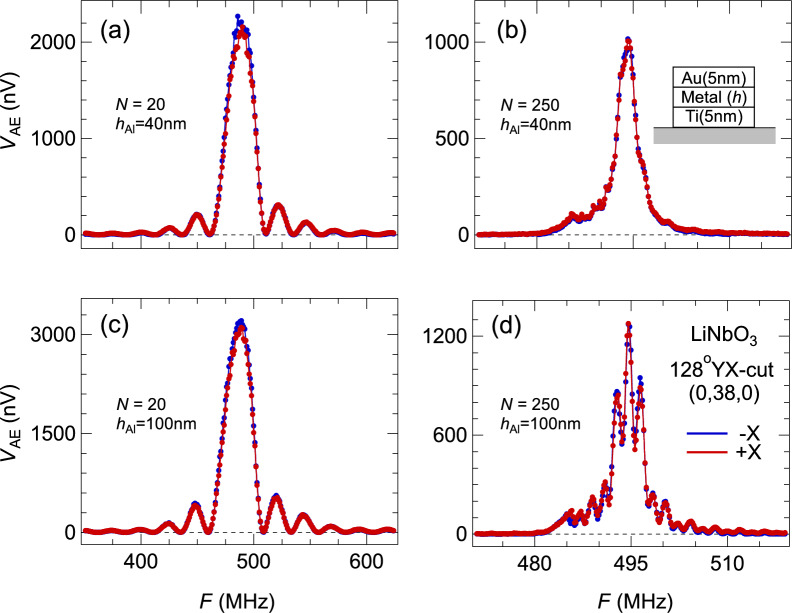
Reciprocal acoustoelectric effect. Measured AE effect from SAW states propagating along $\pm x$-axes of 128YX-cut LiNbO_3_. Each panel of (**a**-**d**) corresponds to a different device with systematically adjusted IDT parameters $N$ and ${h}_{\mathrm{metal}}$. The AE effects have no difference between two opposite directions, thus are reciprocal.

In the configuration of $x$-axis propagation on a 128*YX*-cut substrate, AE measurements along forward ($+x$) and backward ($-x$) directions generate identical frequency profiles (Fig. 2), which verifies the general scheme of our measurements and our devices’ reliability. This reciprocity of SAW along $\pm x$ directions is robust against variations of IDT parameters such as $N$ and the metallic finger height $h$. The frequency spectra’s shapes and magnitudes vary, but the reciprocal relationship is maintained between oppositely propagating SAWs (Fig. 2).

**Fig. 3:**
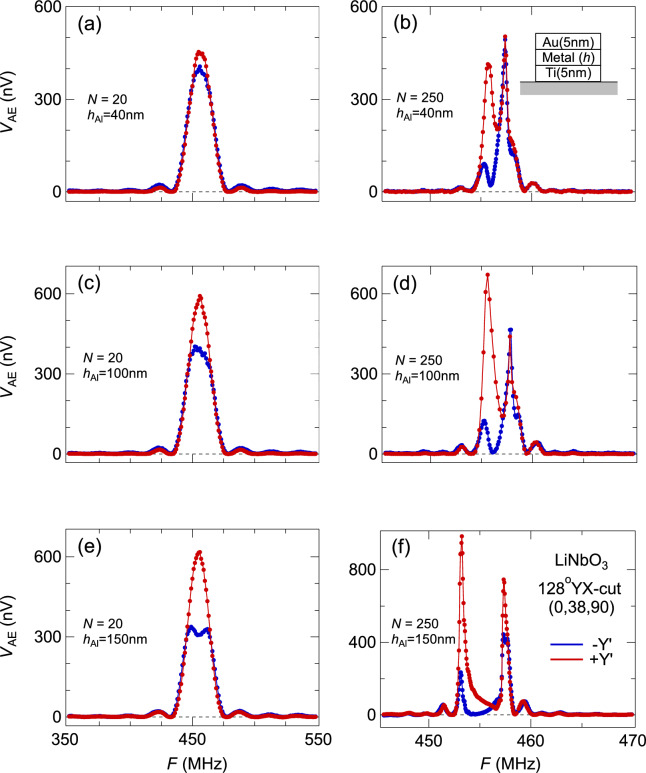
Non-reciprocal acoustoelectric effect. Measured AE effect from SAW states propagating along $\pm y^{\prime}$-axes of 128YX-cut LiNbO_3_. Each panel of (**a**-**f**) corresponds to a different device with varying IDT parameters $N$ and ${h}_{\mathrm{metal}}$. SAW states propagating along opposite directions (red and blue curves) demonstrate the non-reciprocity; their difference grows with increasing amount of either $N$ or ${h}_{\mathrm{metal}}$.

On the other hand, when SAW is propagating along certain directions, such as the in-plane $y^{\prime}$ direction of the 128*YX*-cut substrate (Fig. 3), our measured AE voltages ${V}_{\mathrm{AE}}$ from oppositely propagating SAWs demonstrate a significant difference in their amplitudes, well beyond measurement uncertainty. The ${V}_{\mathrm{AE}}$ difference is enhanced with an increased thickness ${h}_{\mathrm{metal}}$ of metallic fingers (Fig. 3). For devices with a low number of fingers ($N\sim$ 20), the spectral difference resides at the central frequency. However, the difference is shifted sideways when *N* becomes large, and the line shape emerges as two Lorentzian peaks separated in frequency; the ${V}_{\mathrm{AE}}$ difference is more prominent in one (lower) peak of the two (Fig. 3).

In the literature, measured non-reciprocal AE effect has often been attributed to properties of the thin film under study^11,15^. Here, we have used trivial, non-magnetic and polycrystalline metal films that are identical for different reciprocal behaviors in both Figs. 2 and 3. Therefore, there is a clear scenario of non-reciprocal surface acoustic waves because of geometrical configurations relative to the substrate, which in turn leads to non-reciprocal AE effect.

A traveling SAW state necessarily satisfies the time reversal symmetry as a charge phenomenon (Supplementary Materials). The non-reciprocity between strengths of oppositely propagating SAW states thus originates from the stage where SAWs are generated at the interdigital transducer towards both directions, with multiple scattering an integral component of the process. The positive correlation between spectral weight difference and loaded mass (Fig. 3) confirms the mechanical reflection’s influence on the non-reciprocity. The experimental non-reciprocal AE effect thus is closely related to the NSPUDT effect from the SAW device literature.

### Symmetry elements of reciprocal SAW

**Fig. 4:**
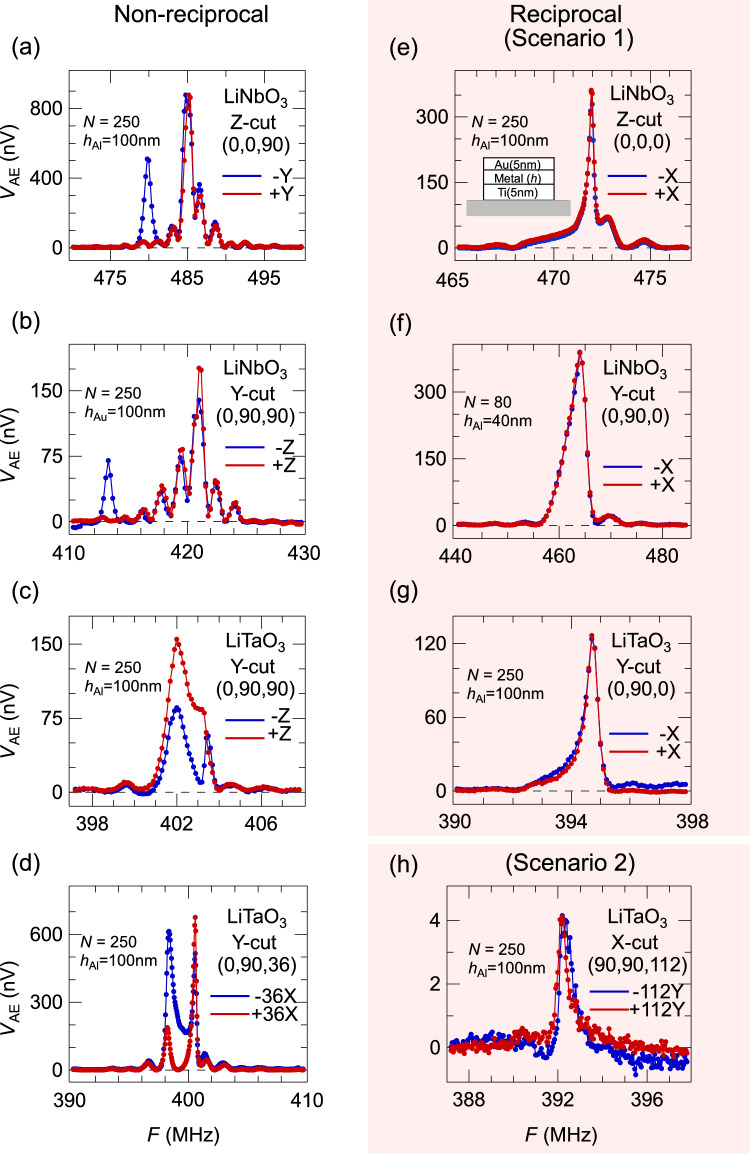
Reciprocity based on choices of the substrate and propagation direction. (**a-h**) Eight devices with different choices of substrate, surface normal, and propagation direction are tested. Both large $N$ and ${h}_{\mathrm{metal}}$ are employed for each device to amplify the potential non-reciprocity of the oppositely propagating SAWs. Panels a-d demonstrate the non-reciprocal AE effect, while panels e-g and h demonstrate reciprocal scenarios of type (1) and (2) respectively. All configurations and substrates were chosen without concerns of beam steering of SAW states^23^.

Here we focus on the LiNbO_3_ and LiTaO_3_ family of SAW substrate materials to experimentally examine the relationship between substrate symmetry and reciprocal/non-reciprocal AE effects. In the piezoelectric phase at room temperature, these crystals belong to the same space group (*R*3c, #161), with non-centrosymmetric point group *3m* operations generated by a mirror $y$-$z$ plane and a threefold rotation axis along $z$. Most of the devices listed in Fig. 4 have both a large $N$ and a large mass load on electrodes to enhance the existence of non-reciprocity. Through Figs. 2–4, ten different configurations are evaluated, with five cases of reciprocal and non-reciprocal AE effects respectively.

All cases of reciprocal AE effects in Figs. 2 and 4 can be attributed to either of the two scenarios: (1) the SAW propagation direction $\widehat{{\boldsymbol{m}}}$ is perpendicular to a mirror plane of the half-space (*e.g*. along the $x$-axis when the axis is within the surface), regardless of the surface normal direction $\widehat{{\boldsymbol{n}}}$ of the substrate, and (2) the substrate surface normal $\widehat{{\boldsymbol{n}}}$ and propagation direction $\widehat{{\boldsymbol{m}}}$ are interchanged from configurations of scenario (1). When $\widehat{{\boldsymbol{n}}}$ is parallel to an axis such as the $x$-axis, the substrate surface, *e.g.* the $y$-$z$ plane, is no longer a mirror plane of the half-space. Yet the propagation direction $\widehat{{\boldsymbol{m}}}$ can be along any direction within the surface plane and the reciprocity is still preserved. The scenario (1) is justified as two oppositely propagating SAW states can be related by the mirror plane as images of each other; the mirror operation remains a proper symmetry element for the half space. On the other hand, for the second scenario, there does not exist any symmetry operation that directly relates the two oppositely propagating SAW states.

The second scenario for reciprocal SAW states appears random and was uncharacterized in Refs.^2,18^. Practically, there exist only a few stable SAW propagation directions without beam steering to make real devices with the $x$-cut substrates of both LiNbO_3_ and LiTaO_3_^23,24^. However, the one-to-one correspondence between configurations of scenarios (1) and (2) provides the missing piece to understand the full symmetry condition for reciprocal SAW states. We note that a SAW state can be constructed from eigenstate solutions of an 8×8 Stroh’s tensor equation $\aleph{\boldsymbol{\xi}}=p{\boldsymbol{\xi}}$ (Supplementary Materials)^19,25,26^. Here the $\aleph$ matrix is fully specified by vectors $\widehat{{\boldsymbol{m}}}$ and $\widehat{{\boldsymbol{n}}}$, and substrate property tensors of elastic constants ${C}_{ijkl}$, piezoelectric constants ${e}_{ijk}$, and dielectric permittivity ${\varepsilon }_{ij}$. ${p}_{\alpha } (\alpha =1,2,\dots , 8)$ are eigenvalues. The eight-component eigenvectors ${{\boldsymbol{\xi}}}^{T}=\left({\boldsymbol{A}},\phi ,{\boldsymbol{L}},{D}_{n}\right)$ are composed of the displacement vector ${\boldsymbol{A}}$, the traction vector ${\boldsymbol{L}}$, the electrical potential $\phi$, and the normal component of the electric displacement field ${D}_{n}$. As the 8×8 tensor equation is transformed into an eigenvalue equation of ${p}_{\alpha }$ (Equation S7 of the Supplementary Materials), $\widehat{{\boldsymbol{m}}}$ and $\widehat{{\boldsymbol{n}}}$ appear as structurally symmetric components. Interchanging $\widehat{{\boldsymbol{m}}}$ and $\widehat{{\boldsymbol{n}}}$ would cause differently valued matrices, but two matrices still have the same matrix structure as of vanishing and non-vanishing components (Supplementary Materials). So the one-to-one correspondence in choices of ($\widehat{{\boldsymbol{m}}}$, $\widehat{{\boldsymbol{n}}}$) between scenarios (1) and (2) is rooted in the symmetry of $\aleph$ matrix equation for the ($\widehat{{\boldsymbol{m}}}$, $\widehat{{\boldsymbol{n}}}$) pair and extends well beyond the point group symmetry-based arguments in Refs.^2,18–21^. Indeed, the ($\widehat{{\boldsymbol{m}}}$, $\widehat{{\boldsymbol{n}}}$) interchangeability in the $\aleph$ matrix equation acts as a hidden symmetry to protect scenario (2).

Our experimental results of reciprocal AE effects in LiNbO_3_ and LiTaO_3 _match with theoretical cases (1) and (3) described in Eq. 24 of Ref.^19^, although the emphasis of symmetry differ. Eq. 24 of Ref.^19^ also listed another two cases (2) and (4) for substrates such as quartz of point group *32*. These two cases also have a one-to-one correspondence that reflects the interchangeability between $\widehat{{\boldsymbol{m}}}$ and $\widehat{{\boldsymbol{n}}}$. Here, the symmetry element is an even-fold rotation axis, when it is perpendicular to the substrate surface. However, when the axis is parallel to the surface, the even-fold axis is no longer a good symmetry element. We again have a situation that the symmetry of Stroh’s equation protects the SAW reciprocity in case (2) of Ref.^19^ while the symmetry element of the lattice point group does not apply at the surface.

With our understanding of the reciprocal SAW states, it is possible to reexamine reported non-reciprocal cases in the literature. In Ref.^15^, it was clear that the SAW states propagated along the $z$-axis of $Y$-cut LiNbO_3_, which is a direction one would expect non-reciprocal SAW states in the substrate. Their understanding of (La,Ca)MnO_3_thus likely requires a re-examination. On the other hand, in Ref.^16^, with a substrate of 128^o^ $YX$-cut LiNbO_3_ and a SAW speed of 3980 m/s, the propagation direction was along the $x$-axis^27^. The observed non-reciprocity should be due to device imperfection and other sample distance effects as the authors stated^16^.

### Direct measurement of the non-reciprocal AE effect

**Fig. 5:**
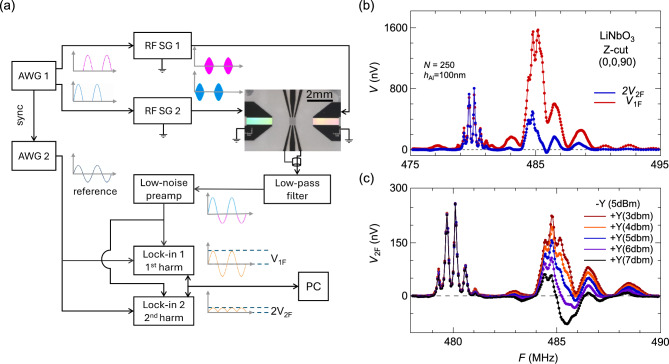
Direct AC harmonic probe of the non-reciprocal AE effect. (**a**) A schematic circuitry to isolate the non-reciprocal AE effect (Methods). The primary frequency signal ${V}_{1\mathrm{F}}$ and second harmonic signal ${V}_{2\mathrm{F}}$ are separately extracted, and ${V}_{1\mathrm{F}}$ should be compared to $2{V}_{2\mathrm{F}}$ based on the measurement scheme. (**b**) A comparison of measured ${V}_{1\mathrm{F}}$ and ${V}_{2\mathrm{F}}$ from a device of identical condition to that of Fig. 4a, using equal input power of 7 dBm on two sides. ${V}_{1\mathrm{F}}$ demonstrate a double-Lorentzian shape with two regions of interest at 480 and 485 MHz. (**c**) With input RF powers fixed along the $-y$ direction and varied along the $+y$ direction, the spectral weight of ${V}_{2\mathrm{F}}$ remains constant at 480 MHz but varies around a non-zero value at 485 MHz. This indicates a complete unidirectional behavior in the 480 MHz region with no spectral weight for the SAW propagating along the $+y$ direction.

Our AC lock-in technique (Methods) can directly extract the non-reciprocal AE effect, using a configuration of a thin metal film between two IDTs (Fig. 5a). Here the AC modulated radio frequency signals are injected alternatively on two IDTs to excite oscillating SAW states in the substrate and both the first and second harmonic components of the AE response are measured (Fig. 5). As alternatively propagating SAWs create oscillating acoustoelectric potentials, the AC signal at the primary frequency represents the averaged AE effect. The non-reciprocal part of the AE effect can be regarded as a unidirectional voltage^11^; its modulation now has the second harmonic frequency (Fig. 5a).

In Fig. 5b, we demonstrate both the primary and second harmonics of the AE voltage. With many IDT pairs *N*=250 and a large electrode mass load ${h}_{\mathrm{Al}}$ = 100 nm, the exemplifying device has a double-Lorentzian profile in the first harmonic AE signal ${V}_{1\mathrm{F}}$ (red curve in Fig. 5b), which contrasts the single-peak spectra of reciprocal AE effects. We choose the substrate geometry with SAW propagating along the *y*-axis of a $z$-cut LiNbO_3_ plate, which corresponds to the case study in Fig 4a of a single IDT and two acoustoelectric measurement pads. ${V}_{1\mathrm{F}}$ in Fig. 5b is very similar to the average of two oppositely traveling curves in Fig. 4a, except a few extra oscillations on top of the spectral weight at 480 MHz that is the triple-pass SAW interference pattern. The overall consistency between Fig. 5b and Fig. 4a justifies our AC lock-in scheme of alternating SAW propagation in Fig. 5a.

The second harmonic acoustoelectric voltage ${V}_{2\mathrm{F}}$ reveals the difference between two oppositely traveling SAW signals. Both ${V}_{1\mathrm{F}}$ and ${V}_{2\mathrm{F}}$ in Fig. 5b have spectral weights at 480 and 485 MHz, but of different proportions. $2{V}_{2\mathrm{F}}$ and ${V}_{1\mathrm{F}}$ have identical amplitudes at 480 MHz, indicating one of two SAW states is effectively zero as demonstrated in Fig. 4a. For devices with two IDTs, there could exist physical differences between IDTs because of variations during the microfabrication process^16^. This can lead to non-zero ${V}_{2\mathrm{F}}$ because of different RF power conversions to sound waves. In Fig. 5c, we vary the power injected into the IDT for the $+y$ propagation, while keep the power injected into the IDT for the $-y$ propagation constant (Fig. 5c). The 480 MHz feature of ${V}_{2\mathrm{F}}$ remains constant, again confirming its origin from a single SAW propagating towards the $-y$ direction.

The simple expression of ${r}^{(\pm )}$ treats electric ${R}_{e}^{\left(\pm \right)}$ and mass ${R}_{m}^{(\pm )}$ loading independently in the literature^2^, and only the mechanical term was regarded to contribute to the NSPUDT effect. From our measurements in Figs. 3f, 4a-4d, and 5b, non-reciprocity behavior also exists at the high-frequency peak, which is regarded as only under the influence of electric reflection^2^. This non-reciprocity at the high frequency peak is especially clear in the second harmonic amplitude ${V}_{2\mathrm{F}}$ at 485 MHz in Fig. 5c. When the mechanical reflection is strong, both loading effects are no longer small perturbative corrections and independent to each other, so the simple approximation of ${r}^{(\pm )}$ breaks down.

## Discussion

Stroh’s $\aleph$-matrix equation of motion is based on the generalized Hooke’s law of linear response, including the piezoelectric effects^19,25,26^, but does not take the reflection effect into consideration. As $\aleph$ is a real matrix, its eigenvalues and eigenstates both appear in pairs of complex conjugates, ${p}_{\gamma }={p}_{\gamma +4}^{*} (\gamma =\mathrm{1,2},\mathrm{3,4})$ and ${{\boldsymbol{\xi}}}_{\gamma }={{\boldsymbol{\xi}}}_{\gamma +4}^{*}$, representing two oppositely propagating *bulk* acoustic waves (Supplementary Materials). The surface acoustic wave state is constructed from eigenstates of those ${p}_{\alpha }$ with an imaginary component that would lead to a decay into the depth, and with coefficients constrained by the surface boundary conditions of both traction and the electric displacement field. All constructed SAW states by the $\aleph$-matrix equation are time reversal symmetric, but SAW states that are eventually reciprocal or non-reciprocal in real devices have eigenvalues and eigenstates of two different structures (Supplementary Materials). When either $\widehat{{\boldsymbol{m}}}$ or $\widehat{{\boldsymbol{n}}}$ is perpendicular to a mirror plane of the bulk LiNbO_3_ (such as along the $x$-axis), the $\aleph$ matrix can be transformed into a block matrix composed of four 4 $\times$ 4 matrices with null matrices along the diagonal. With additional real and symmetric properties of off-diagonal matrices, this form can only lead to either purely real or purely imaginary eigenvalues in conjugated pairs, and in turn, individual eigenstates ${\boldsymbol{\xi}}$ with components of phase differences fixed at multiples of $\pi /2$ for the latter case (Supplementary Materials). Conversely, when neither $\widehat{{\boldsymbol{m}}}$ nor $\widehat{{\boldsymbol{n}}}$ is perpendicular to a mirror plane, $\aleph$ is no longer traceless and cannot be reduced into a simple form. Each eigenvalue can thus have both real and imaginary components. Components of each eigenstate would have phase differences not bound to multiples of $\pi /2$ (Supplementary Materials). For those states, multiple reflections at IDTs would induce observable NSPUDT effects^2^.

Ref.^19^ approaches reciprocal SAW states from a different perspective of standing waves. As a resonance in the periodic IDT structure, a SAW state can have its frequency determined by either zeros or poles of the harmonic admittance under *open-* or *short-circuit* configurations respectively^19,28,29^. Ref.^19^ argues that when frequency positions of a zero and a pole are degenerate, there would simultaneously be no current nor voltage on the electrodes^21^, signaling the presence of a pure standing wave state. Such a standing wave state is made of oppositely traveling SAW states of equal amplitudes, which are reciprocal. Instead of analytically solving for eigenstates of the $\aleph$ matrix, the admittance was constructed in Ref.^19^ by numerical solutions through coupling-of-modes equations that include the multiple reflection process. While the approach in Ref.^19^ reveals previously unnoticed configurations in Ref.^18^, it did not offer a clear understanding of symmetry effects and the connection between two scenarios. More specifically, we notice the determinant ${\Delta }^{(q)}$in Eq. 22 of Ref.^19^ would still have both zero and non-zero values for $\aleph$ matrix eigenstates, which would all have the time reversal symmetry in the absence of the multiple reflection. This parameter ${\Delta }^{(q)}$ is thus not associated with standing wave states *in general* but rather represents an alternative statement about the two types of $\aleph$ matrix eigenstates.

Our listed scenario (1) has a clear symmetry element that is applicable to the macroscopic half-space. In substrates of point group *3m*, it is the mirror plane perpendicular to the substrate surface; for quartz of point group *32*, it is a two-fold rotational axis perpendicular to the substrate surface. In general, a rotational axis perpendicular to the substrate surface would remain as a valid symmetry operation for the half-space as it is for the bulk. As a Rayleigh surface acoustic wave only depends on coordinates ${x}_{1}$ and ${x}_{3}$ along $\widehat{{\boldsymbol{m}}}$ and $\widehat{{\boldsymbol{n}}}$ respectively but not the transverse coordinate ${x}_{2}$ (Supplementary Materials), either symmetry operation of the mirror plane perpendicular to $\widehat{{\boldsymbol{m}}}$ or a two-fold rotational axis parallel to $\widehat{{\boldsymbol{n}}}$ gives the same transformation of ${x}_{1}\to -{x}_{1}$ and ${x}_{3}\to {x}_{3}$, while the change of ${x}_{2}\to \pm {x}_{2}$ is irrelevant to SAW states. Either of these symmetry elements can connect two oppositely propagating SAW states to preserve the reciprocity.

Scenario (2) does not have the global symmetry protection as that of scenario (1). However, for two configurations using either $\widehat{{\boldsymbol{m}}}$ or $\widehat{{\boldsymbol{n}}}$ as the surface norm and the other as the SAW propagation direction, they share similarly structured $\aleph$ tensors because of the construction of Stroh’s equation. We note that for the propagation of SAW, the substrate does not need to be piezoelectric; the piezoelectric material is only necessary for the generation of SAWs by IDTs. So both the original 6 $\times$ 6 and the extended 8 $\times$ 8 Stroh’s matrix equations^25,26^ possess the symmetry structure that leads to the interchangeability between $\widehat{{\boldsymbol{m}}}$ and $\widehat{{\boldsymbol{n}}}$. This symmetric structure becomes apparent in the direct expression of the equation of motion. As the basis of Stroh’s 6 $\times$ 6 tensor, two phenomena, the continuity of media $\partial {\sigma }_{ij}/\partial {x}_{i}=\rho {\partial }^{2}{u}_{j}/\partial {t}^{2}$, and the Hooke’s law ${\sigma }_{ij}={C}_{ijkl}\partial {u}_{k}/\partial {x}_{l}$, where ${\sigma }_{ij}$ is the stress tensor, ${u}_{i}$ is the SAW displacement, and ${x}_{i}$ ($i=\mathrm{1,2},3$) the real space coordinates, combine to form the equation of motion ${C}_{ijkl}{\partial }^{2}{u}_{k}/\partial {x}_{i}\partial {x}_{l}=\rho {\partial }^{2}{u}_{j}/\partial {t}^{2}$^1,25^. For Stroh’s 8 $\times$ 8 tensor that includes the piezoelectric tensor ${e}_{ijk}$, the equation of motion is extended to ${C}_{ijkl}{\partial }^{2}{u}_{k}/\partial {x}_{i}\partial {x}_{l}+{e}_{ijk}{\partial }^{2}\phi /\partial {x}_{i}\partial {x}_{k}=\rho {\partial }^{2}{u}_{j}/\partial {t}^{2}$^21,26^. In a coordinate system of $\widehat{{\boldsymbol{m}}}$, $\widehat{{\boldsymbol{n}}}$, and $\widehat{{\boldsymbol{m}}}\times \widehat{{\boldsymbol{n}}}$, a Rayleigh type SAW has only $\widehat{{\boldsymbol{m}}}$ and $\widehat{{\boldsymbol{n}}}$ components that are independent of $\widehat{{\boldsymbol{m}}}\times \widehat{{\boldsymbol{n}}}$. As a part of the equation of motion, the off-diagonal shear component of the strain tensor ${s}_{mn}={s}_{nm}=\partial {u}_{m}/\partial {x}_{n}+\partial {u}_{n}/\partial {x}_{m}$ is by definition symmetric^1^. So both equations of motion are essentially two-dimensional with displacements ${u}_{m}$ and ${u}_{n}$ treated in a structurally symmetrical manner (despite coefficients involving the phonon speed can be of different values along two directions). Physically, each volumetric element of a Rayleigh SAW state is simultaneously involved in a compressional motion along $\widehat{{\boldsymbol{m}}}$ and a shear motion along $\widehat{{\boldsymbol{n}}}$. As $\widehat{{\boldsymbol{m}}}$ in scenario (1) becomes $\widehat{{\boldsymbol{n}}}$ in scenario (2), and vice versa, the symmetric mathematical structure assures the one-to-one correspondence between the two.

A salient feature of Stroh’s equation is that there exists no intrinsic length scale, or rather that, while the speed of SAW is subsonic to the bulk speed of sound, it remains wavelength independent. The equation of motion is established over the unit cell spacing that is much shorter than the typical SAW wavelength. At the nanometer scale, the bulk nature is fully preserved for the equation of motion. Only approaching the substrate surface, this perspective of local bulk property would encounter a potential conflict, which is resolved by the boundary condition that requires both the traction ${\boldsymbol{L}}$ and electric displacement field component ${D}_{n}$ to approach zero at the surface. The point group symmetry element for scenario (1) holds across the global scale exceeding the wavelength distance, matching to the perspective of hydrodynamic continuity. The mathematical symmetry in the equation of motion holds locally at the nanometer scale to ensure scenario (2). Through this perspective, scenario (2) can be regarded as relying on a hidden, local protection that is independent of the substrate symmetry. Indeed, such a one-to-one correspondence between two configurations marked by a pair of $\widehat{{\boldsymbol{m}}}$ and $\widehat{{\boldsymbol{n}}}$ exist in general and would remain even if the SAW states are of the non-reciprocal type.

SAW states have been suggested for skyrmion creation and manipulation^5,30,31^, as several groups utilize four IDTs in a cross pattern to trap magnetic excitations by nodes and antinodes of the standing SAW waves. However, if SAW states are not reciprocal, a standing wave would be unstable and drifting. Our study suggests for substrates such as 128^o^ $YX$-cut LiNbO_3_^31^, some of the IDTs should be separately driven, in a fashion similar to the scheme in Fig. 5a, in order to create matching SAW amplitudes. This instance of reciprocity as a hidden assumption is rather common in many SAW applications.

## Methods

### Microfabrication of SAW devices

Our SAW devices to explore the AE effect are composed of two parts, an interdigital transducer to generate the propagating SAW states and a thin metal film to measure the AE effect; both are built on top of a piezoelectric substrate, of either LiNbO_3_ or LiTaO_3_. Substrates of different cuts (orientations) were purchased from several commercial vendors. All SAW devices were fabricated in a cleanroom using maskless photolithography with a minimum feature size of 2 $\mu \mathrm{m}$. The fabrication process consisted of three lithographic stages, first the IDT(s), then the metal film, and finally Au electrode pads for wire bonding. All metals were deposited inside an electron-beam evaporation system. The IDTs are built with parameters listed in Figs. 1-5, first with a 5nm Ti adhesive layer, then either an Al or Au layer as the bulk of IDT, then a topping layer of 5nm Au for the protection of Al oxidation. The thickness of the metal film should be about 3–7 nm, in order to generate a strong AE effect^32^. We chose the thin metal film to be made of non-magnetic elemental metals in a bi-layer structure of Ti (2 nm) and Au (3 nm). The electrode pads are made of Au of 100 nm thickness. To avoid edge effects of the device that reflects waves back towards the IDT, small droplets of superglue were placed on the SAW pathway outside of the device region to provide effective damping and dissipation. Each piezoelectric substrate is anchored to a printed circuit board of planar RF waveguides by supergluing its corners; these two are electrically connected by 25 $\mu$ m diameter Au wires through wire bonding. The printed circuit board uses surface-mount SMA connectors to further connect to RF power sources, and to VNA devices for the initial device characterization. The AE voltages in the AC frequency range are picked up through normal pin connectors.

### AC lock-in measurements for the acoustoelectric effect

Many groups measured the AE effect using a DC electrical current source. Here we measure the AE effect using the lock-in technique in the audio-frequency range in order to suppress the ground current effect and achieve a high sensitivity of signals below 5 nV (Fig. 4h).

Fig. 1a outlines a single, symmetric IDT measurement scheme for reciprocity, featuring the IDT as the identical source for two oppositely propagating SAWs. Comparing to designs with two IDTs, it avoids microfabrication differences between two sources. In Fig. 1a, an arbitrary waveform generator (SDG2082X, Siglent Technologies Co.) provides an AC frequency sine wave time series (~49 Hz), which in turn amplitude modulates a RF signal generator (SG386, Stanford Research Systems) for the excitation of the IDT. We typically use a +7dBm (~5mW) input RF power for the SAW excitation, which is the maximum power of the signal generator. From the literature a 21dBm (~158mW) power injection would increase the device temperature by ~10 K^33^, so our input power is not expected to generate noticeable heating effect in comparison to our measurement temperature of 295 K. Given the weak AE signal, a home-built low-pass filter using commercial components with a cut-off frequency of 500 kHz (ELKEA103FA, Panasonic Inc.) was first applied to suppress the RF noise and prevent preamplifier saturation. The low pass filtered signal is further amplified by a low noise pre-amplifier (SRS560, Stanford Research Systems) with a typical amplification factor of 200$\times$. Both the pre-amplified AE signal and the reference sine wave signal from the original arbitrary waveform generator are passed to the lock-in amplifier (SR830, Stanford Research Systems), which extracts the AE voltage from the second harmonic channel of the reference sine wave.

Fig. 5a outlines the scheme to directly measure the non-reciprocal AE signal using the AC-frequency lock-in approach. Here two IDTs are utilized to provide alternatively and oppositely propagating SAWs towards the conductive pad in the center. The waveform evolution at each stage is outlined in Fig. 5a. As each IDT is driven by RF signals with a modulated amplitude of a half sine wave, the AE voltage that is proportional to the power of RF signal is composed of a series of odd harmonics of the primary AC frequency, but not the even harmonics. On the other hand, non-reciprocal behavior would lead to even harmonic components of measurable intensities. To make a fair comparison, one would compare ${V}_{1\mathrm{F}}$ with ${2V}_{2\mathrm{F}}$ (Fig. 5a), as ${V}_{1\mathrm{F}}=({V}_{\mathrm{L}}+{V}_{\mathrm{R}})/2$ and $2{V}_{2\mathrm{F}}=\left|{V}_{\mathrm{L}}-{V}_{\mathrm{R}}\right|/2$, where ${V}_{\mathrm{L}}$ and ${V}_{\mathrm{R}}$ are absolute values of the AE amplitudes from left and right IDTs respectively.

The scheme in Fig. 5a takes into consideration of practical complications. In reality, two IDTs might not be perfect copies of each other but can have finite differences during micro-fabrication^16^. In our scheme, each IDT is driven by an independent RF power source, and the balance of power input can be adjusted between two directions. A varying power study is exemplified in Fig. 5c and can serve to demonstrate both the complete and incomplete NSPUDT (unidirectional) behavior at ~480 MHz and ~485 MHz respectively.

## Supplementary Information

Supplementary Information.

## Data Availability

All study data are included in the article and/or supplementary materials.
