# Supplementary material for: Nanoscale symmetry protection of the reciprocal acoustoelectric effect
Source: Sci Rep. 2026 Feb 6;16:7637. doi: 10.1038/s41598-026-38987-6 (PMC12936158; doi:10.1038/s41598-026-38987-6)
Supplement: Supplementary file 1 — Supplementary Information. [file 41598_2026_38987_MOESM1_ESM.pdf]

Supplementary Materials for:

Nanoscale symmetry protection of the reciprocal acoustoelectric effect

Sandeep Vijayan[1], Stephan Suffit[1], Scott E. Cooper[1], Yejun Feng[1,*]

[1]Okinawa Institute of Science and Technology Graduate University, Onna, Okinawa 904-0495, Japan

*Corresponding author: yejun@oist.jp

Supplementary note: Surface acoustic waves in a substrate of point group *3m*

1. SAW states as eigenstate solutions of a matrix formalism

In 1960's, Stroh [25] provided a formalism to represent the elasto-dynamic equations of an anisotropic solid as an eigenvalue problem of a 6×6 matrix, known as the Stroh matrix. The matrix only depends on the elasticity tensor, the velocity of the wave in the medium, and the density of the medium. This formalism emerges from a pair of vector equations that couple two three-dimensional vectors, namely the displacement vector and the traction vector. With the presence of both vectors, a six-dimensional state vector can be formed, which serves as the eigenvector of the Stroh matrix. Lothe and Barnett [26] further improved the formalism by incorporating the electrostatic equations along with the elasto-dynamic equations, assuming a semi-infinite piezoelectric solid. This addition morphs the 6×6 Stroh matrix into an 8×8 matrix, which also requires the extra knowledge of the piezoelectric tensor and the permittivity tensor for its formation. Each eight-dimensional eigenvector is composed of the displacement vector $\boldsymbol{A}$, electrostatic potential ϕ, traction vector $\boldsymbol{L}$, and the normal component of the displacement field D_n.

Here we summarize the general formalism. We assume that a semi-infinite piezoelectric medium has a surface normal along the unit vector $\hat{\boldsymbol{n}}$ and the wave is propagating along the unit vector $\hat{\boldsymbol{m}}$ within the surface (Fig. 1). So, the SAW state can be treated as an eigenstate problem as:

$$\widehat{\boldsymbol{N}}\boldsymbol{\xi}_\alpha = p_\alpha \boldsymbol{\xi}_\alpha, \qquad \alpha = 1,2,\dots,8. \tag{S1}$$

Here, p_α are eigenvalues, $\boldsymbol{\xi}_\alpha$ are eigenfunctions, and $\widehat{\boldsymbol{N}}$ is an 8×8 matrix defined by four blocks of 4×4 matrices as:

$$\widehat{\boldsymbol{N}} = -\begin{bmatrix} (nn)^{-1}(nm) & (nn)^{-1} \\ (mn)(nn)^{-1}(nm) - (mm) & (mn)(nn)^{-1} \end{bmatrix}, \tag{S2}$$

with the matrices $(nn), (mn), (nm)$ and (mm) defined in the following manner [24],

$$(ab) = \begin{bmatrix} a_k c_{k11l} b_l - \rho v^2 a_k m_k m_l b_l & a_k c_{k12l} b_l & a_k c_{k13l} b_l & a_k e_{l1k} b_l \\ a_k c_{k21l} b_l & a_k c_{k22l} b_l - \rho v^2 a_k m_k m_l b_l & a_k c_{k23l} b_l & a_k e_{l2k} b_l \\ a_k c_{k31l} b_l & a_k c_{k32l} b_l & a_k c_{k33l} b_l - \rho v^2 a_k m_k m_l b_l & a_k e_{l3k} b_l \\ a_k e_{k1l} b_l & a_k e_{k2l} b_l & a_k e_{k3l} b_l & -a_k \varepsilon_{kl} b_l \end{bmatrix}.$$

Here, $\boldsymbol{c}, \boldsymbol{e}$ and $\boldsymbol{\varepsilon}$ are the elasticity, piezoelectric, and permittivity tensors respectively; v is the phase velocity of the surface acoustic wave along $\hat{\boldsymbol{m}} = (m_1, m_2, m_3)$, ρ is the mass density of the material, and the summation runs over indices k and l from 1 to 3. At this stage, the coordination system (1, 2, 3) represents the three-dimensional space and can be rather general as long as the three axes are orthogonal. Both $\hat{\boldsymbol{m}}$ and $\hat{\boldsymbol{n}}$ vectors are projected onto this coordinate system.

From Equation S2, we define,

$$\hat{\boldsymbol{A}} = -(nn)^{-1}(nm),$$
$$\hat{\boldsymbol{B}} = -(nn)^{-1},$$
$$\hat{\boldsymbol{C}} = -(mn)(nn)^{-1}(nm) + (mm). \tag{S3}$$

Then Equation S2 can be expressed as,

$$\hat{\boldsymbol{N}} = \begin{bmatrix} \hat{\boldsymbol{A}} & \hat{\boldsymbol{B}} \\ \hat{\boldsymbol{C}} & \hat{\boldsymbol{A}}^{\boldsymbol{T}} \end{bmatrix}. \tag{S4}$$

The eigenvalue problem becomes:

$$\det(\hat{\boldsymbol{N}} - p_\alpha \hat{\boldsymbol{I}}_8) = 0. \tag{S5}$$

Using Schur's complement, we can rewrite Equation S5 as,

$$\det(\hat{\boldsymbol{A}} - p_\alpha \hat{\boldsymbol{I}}_4)\det\left(\hat{\boldsymbol{A}}^{\boldsymbol{T}} - p_\alpha \hat{\boldsymbol{I}}_4 - \hat{\boldsymbol{C}}(\hat{\boldsymbol{A}} - p_\alpha \hat{\boldsymbol{I}}_4)^{-1}\hat{\boldsymbol{B}}\right) = 0.$$

Since p_α is the eigenvalue for $\hat{\boldsymbol{N}}$ but not $\hat{\boldsymbol{A}}$, $\det(\hat{\boldsymbol{A}} - p_\alpha \hat{\boldsymbol{I}}_4) \neq 0$. We should have,

$$\det\left(\hat{\boldsymbol{A}}^{\boldsymbol{T}} - p_\alpha \hat{\boldsymbol{I}}_4 - \hat{\boldsymbol{C}}(\hat{\boldsymbol{A}} - p_\alpha \hat{\boldsymbol{I}}_4)^{-1}\hat{\boldsymbol{B}}\right) = 0,$$
$$\Rightarrow \det\left(\hat{\boldsymbol{A}}^{\boldsymbol{T}}\hat{\boldsymbol{B}}^{-\boldsymbol{1}} - p_\alpha \hat{\boldsymbol{B}}^{-\boldsymbol{1}} - \hat{\boldsymbol{C}}(\hat{\boldsymbol{A}} - p_\alpha \hat{\boldsymbol{I}}_4)^{-1}\right)\det(\hat{\boldsymbol{B}}) = 0, \qquad \because \det(\hat{\boldsymbol{B}}) \neq 0$$
$$\Rightarrow \det\left(\hat{\boldsymbol{A}}^{\boldsymbol{T}}\hat{\boldsymbol{B}}^{-\boldsymbol{1}}(\hat{\boldsymbol{A}} - p_\alpha \hat{\boldsymbol{I}}_4) - p_\alpha \hat{\boldsymbol{B}}^{-\boldsymbol{1}}(\hat{\boldsymbol{A}} - p_\alpha \hat{\boldsymbol{I}}_4) - \hat{\boldsymbol{C}}\right)\det\left((\hat{\boldsymbol{A}} - p_\alpha \hat{\boldsymbol{I}}_4)^{-1}\right) = 0,$$
$$\Rightarrow \det\left(p_\alpha^2 \hat{\boldsymbol{B}}^{-\boldsymbol{1}} - p_\alpha(\hat{\boldsymbol{A}}^{\boldsymbol{T}}\hat{\boldsymbol{B}}^{-\boldsymbol{1}} - \hat{\boldsymbol{B}}^{-\boldsymbol{1}}\hat{\boldsymbol{A}}) + \hat{\boldsymbol{A}}^{T}\hat{\boldsymbol{B}}^{-\boldsymbol{1}}\hat{\boldsymbol{A}} - \hat{\boldsymbol{C}}\right) = 0. \tag{S6}$$

Substitute Equations S3 into Equation S6, we get an eigenvalue equation:

$$\det(p_\alpha^2(nn) - p_\alpha((mn) + (nm)) + (mm)) = 0. \tag{S7}$$

With the coefficient matrices all real and symmetric, it is necessary that eigenvalues must be either real or occur in pairs of complex conjugates (p_γ, p_γ^*), with $\gamma = 1{,}2{,}3{,}4$, and the eigenvectors $(\boldsymbol{\xi}_\gamma, \boldsymbol{\xi}_\gamma^*)$ must also occur in complex conjugate pairs.

As an 8×8 matrix, $\hat{\boldsymbol{N}}$ has eight eigenvalues (p_α) and eigenvector $(\boldsymbol{\xi}_\alpha)$, so there are eight modes, defined as:

$$\boldsymbol{\xi}_\alpha(\boldsymbol{r}, t) = \boldsymbol{\xi}_\alpha \exp[i\boldsymbol{k} \cdot (\hat{\boldsymbol{m}} r_1 + p_\alpha \hat{\boldsymbol{n}} r_3 - \boldsymbol{v}t)], \quad \alpha = 1{,}2, \ldots, 8. \tag{S8}$$

Here $\hat{\boldsymbol{m}}$ and $\hat{\boldsymbol{n}}$ define a wave-based coordinate system ($\hat{\boldsymbol{m}}$, $\hat{\boldsymbol{m}} \times \hat{\boldsymbol{n}}$, $-\hat{\boldsymbol{n}}$) for the surface wave states (Fig. 1). $\boldsymbol{r} = (r_1, r_2, r_3)$ is the spatial position.

All eight modes can be divided into two equal sized groups, with eigenvalues of one group always the complex conjugate of the other. The grouping scheme based on complex conjugacy separates waves going to one side ($\boldsymbol{k}$ and $\boldsymbol{v}$) and modes going to the opposite direction ($\boldsymbol{k}' = -\boldsymbol{k}$ and $\boldsymbol{v}' = -\boldsymbol{v}$). For a pair of oppositely propagating waves, we have $\boldsymbol{\xi}_\gamma(\boldsymbol{r}, t) = \boldsymbol{\xi}_\gamma \exp\left[i\boldsymbol{k} \cdot \left(\hat{\boldsymbol{m}} \cdot r_1 + p_\gamma \hat{\boldsymbol{n}} \cdot r_3 - \boldsymbol{v}t\right)\right]$, and

$$\boldsymbol{\xi}_{\gamma+4}(\boldsymbol{r}, t) = \boldsymbol{\xi}_{\gamma+4} \exp\left[-i\boldsymbol{k} \cdot \left(\hat{\boldsymbol{m}} \cdot r_1 + p_\gamma^* \hat{\boldsymbol{n}} \cdot r_3 + \boldsymbol{v}t\right)\right], \quad \gamma = 1{,}2{,}3{,}4.$$

Here we already used $p_{\gamma+4} = p_\gamma^*$, $\boldsymbol{k}' = -\boldsymbol{k}$, and $\boldsymbol{v}' = -\boldsymbol{v}$, and for the amplitude, we have $\boldsymbol{\xi}_\gamma = \boldsymbol{\xi}_{\gamma+4}^*$. The time reversal operation $\mathbf{T}$ transforms a state as $\mathbf{T}\boldsymbol{\xi}_\gamma(\boldsymbol{r}, t) = \boldsymbol{\xi}_\gamma^*(\boldsymbol{r}, -t)$. It is straightforward to verify that $\boldsymbol{\xi}_\gamma^*(\boldsymbol{r}, -t) = \boldsymbol{\xi}_{\gamma+4}(\boldsymbol{r}, t)$. So, the oppositely propagating $\boldsymbol{\xi}_\gamma(\boldsymbol{r}, t)$ and $\boldsymbol{\xi}_{\gamma+4}(\boldsymbol{r}, t)$ are always equivalent under the time reversal operation. This conforms to the time reversal symmetry for a charge system that one propagating mode is simply the time reversed mode of the oppositely propagating one.

Since our analysis is for SAWs, it becomes sufficient to focus on only the modes that correspond to truly complex eigenvalues. This is because those modes with real eigenvalues cannot decay into the bulk, and if included, each will result in a bulk wave.

The normalization of the eigenvectors takes the following appraoch. Based on the form of $\widehat{\boldsymbol{N}}$, we introduce the tensor

$$\widehat{\boldsymbol{T}} = \begin{bmatrix} 0 & 0 & 0 & 0 & 1 & 0 & 0 & 0 \\ 0 & 0 & 0 & 0 & 0 & 1 & 0 & 0 \\ 0 & 0 & 0 & 0 & 0 & 0 & 1 & 0 \\ 0 & 0 & 0 & 0 & 0 & 0 & 0 & 1 \\ 1 & 0 & 0 & 0 & 0 & 0 & 0 & 0 \\ 0 & 1 & 0 & 0 & 0 & 0 & 0 & 0 \\ 0 & 0 & 1 & 0 & 0 & 0 & 0 & 0 \\ 0 & 0 & 0 & 1 & 0 & 0 & 0 & 0 \end{bmatrix},$$

to make the product $\widehat{\boldsymbol{T}}\widehat{\boldsymbol{N}}$ symmetric. Now we rewrite Equation S1 as,

$$\widehat{\boldsymbol{T}}\widehat{\boldsymbol{N}}\boldsymbol{\xi}_\alpha = p_\alpha \widehat{\boldsymbol{T}}\boldsymbol{\xi}_\alpha$$
$$\Rightarrow \boldsymbol{\xi}_\alpha^T \widehat{\boldsymbol{T}}\widehat{\boldsymbol{N}} = p_\alpha \boldsymbol{\xi}_\alpha^T \widehat{\boldsymbol{T}}. \tag{S9}$$

So $\boldsymbol{\xi}_\alpha^T \widehat{\boldsymbol{T}}$ is the left eigenvector of $\widehat{\boldsymbol{N}}$, which allows a normalization condition on the eigenvectors as,

$$\boldsymbol{\xi}_\alpha^T \widehat{\boldsymbol{T}} \boldsymbol{\xi}_\beta = \delta_{\alpha\beta}\ , \qquad \alpha, \beta = 1,2,\dots,8 \tag{S10}$$

The individual mode is characterized as $\boldsymbol{\xi}_\alpha = (A_{\alpha 1}, A_{\alpha 2}, A_{\alpha 3}, \phi_\alpha, L_{\alpha 1}, L_{\alpha 2}, L_{\alpha 3}, D_\alpha)^T$, where $A_{\alpha i}$ are components of the elastic displacement amplitude vector $\boldsymbol{A}_\alpha$, $\boldsymbol{L}_\alpha$ is proportional to the surface traction $\boldsymbol{f}_\alpha$ as $\boldsymbol{L}_\alpha = i\boldsymbol{f}_\alpha/k$, ϕ_α is the electric potential, and D_α is proportional to the normal component of the displacement field $D_{n\alpha}$ as $D_\alpha = iD_{n\alpha}/k$ [19, 26]. All are associated with a given eigenvalue p_α.

From here on, we further analyze the $\widehat{\boldsymbol{N}}$ matrix for conditions leading to reciprocal and non-reciprocal (or symmetrical vs. asymmetrical) SAW states, in relation to the lattice symmetry.

2. Scenarios of reciprocal SAW states

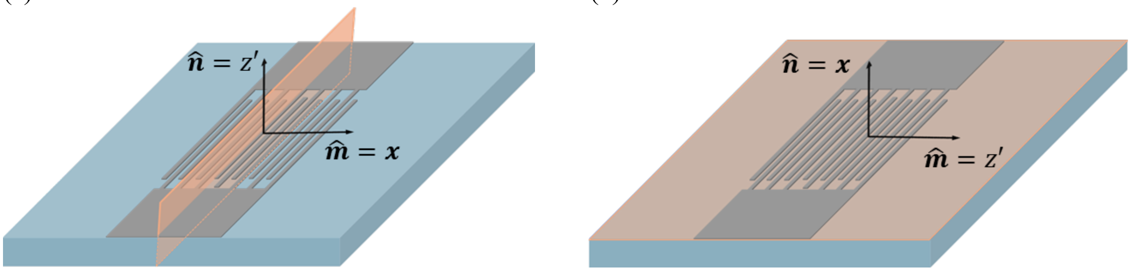

Figure S1: Schematics of reciprocal SAW states. (a) Propagation vector $\widehat{\boldsymbol{m}}$ is along the crystalline x-axis, and an arbitrary direction z' in the y-z plane can be chosen as the substrate surface normal $\widehat{\boldsymbol{n}}$, provided the beam steering effect is not a concern. (b) Conversely, the substrate surface normal $\widehat{\boldsymbol{n}}$ is parallel to the crystalline x-axis and the propagation is along an arbitrary direction z' within the y-z plane. The orange plane represents the mirror y-z plane of the bulk crystal.

In the current study, we have used substrates of $LiNbO_3$ and $LiTaO_3$, which are of the same point group $3m$ and without the inversion symmetry. In this section, we examine all the substrate geometries that have either $\widehat{\boldsymbol{m}}$ or $\widehat{\boldsymbol{n}}$ vectors along the crystalline x-axis, which is perpendicular to the mirror plane as shown in Fig. S1. Instead of using the typical coordinate system ($\widehat{\boldsymbol{m}}$, $\widehat{\boldsymbol{m}} \times \widehat{\boldsymbol{n}}$, $-\widehat{\boldsymbol{n}}$), we express the $\widehat{\boldsymbol{N}}$

matrix in a sample-oriented coordinate system (1, 2, 3) = (x, y', z'). This coordinate system is designed to take the advantage that in this note, we only consider scenarios that the crystalline x-axis is parallel to one of the three orthogonal vectors $\hat{\boldsymbol{m}}$, $\hat{\boldsymbol{n}}$, (discussed here) or $\hat{\boldsymbol{m}} \times \hat{\boldsymbol{n}}$ (discussed in the next section). After the x-axis is matched, the remaining two orthogonal axes are within the crystalline y-z plane and are related to the y and z axes by a rotational transformation. We thus define the remaining two orthogonal axes among $\hat{\boldsymbol{m}}$, $\hat{\boldsymbol{n}}$, and $\hat{\boldsymbol{m}} \times \hat{\boldsymbol{n}}$ as y' and z' to form the (x, y', z') coordinate system. These (x, y', z') coordinates are essentially permutated ($\hat{\boldsymbol{m}}$, $\hat{\boldsymbol{m}} \times \hat{\boldsymbol{n}}$, $-\hat{\boldsymbol{n}}$) coordinates but can better incorporate in the crystalline symmetry. For both cases considered in this section, we have either $\hat{\boldsymbol{m}}||x$ ($\hat{\boldsymbol{n}}||z'$) or $\hat{\boldsymbol{n}}||x$ ($\hat{\boldsymbol{m}}||z'$). They both lead the $\hat{\boldsymbol{N}}$ matrix to a general form as,

$$\hat{\boldsymbol{N}} = \begin{bmatrix} 0 & \times & \times & \times & \times & 0 & 0 & 0 \\ 0 & 0 & 0 & 0 & 0 & \times & \times & \times \\ 1 & 0 & 0 & 0 & 0 & \times & \times & \times \\ 0 & 0 & 0 & 0 & 0 & \times & \times & \times \\ \times & 0 & 0 & 0 & 0 & 0 & 1 & 0 \\ 0 & \times & \times & \times & \times & 0 & 0 & 0 \\ 0 & \times & \times & \times & \times & 0 & 0 & 0 \\ 0 & \times & \times & \times & \times & 0 & 0 & 0 \end{bmatrix}. \tag{S11}$$

Here, × represents a term that can have a non-zero value based on current $\hat{\boldsymbol{m}}$ and $\hat{\boldsymbol{n}}$ orientaions and numerical values of the substrate's property tensors.

The form of $\hat{\boldsymbol{N}}$ in Equation S11 can lead to two 4×4 null matrices along the diagonal. As eingenvalues remain invariant upon a permutation transformation of the matrix, we move row 1 to row 8 and the corresponding column 1 to column 8 in the matrix expression of S11, this results in a new matrix $\widehat{\boldsymbol{N}'}$ with a more compact form,

$$\widehat{\boldsymbol{N}'} = \begin{bmatrix} \hat{\boldsymbol{0}} & \widehat{\boldsymbol{B}'} \\ \widehat{\boldsymbol{C}'} & \hat{\boldsymbol{0}} \end{bmatrix}, \tag{S12}$$

where $\hat{\boldsymbol{0}}$ is a null 4×4 matrix and $\widehat{\boldsymbol{B}'}, \widehat{\boldsymbol{C}'}$ are non-zero 4×4 matrices. From S4, one can observe that both $\widehat{\boldsymbol{B}'}$ and $\widehat{\boldsymbol{C}'}$ are real and symmetric tensors. Since eigenvectors of $\widehat{\boldsymbol{N}'}$ and $\hat{\boldsymbol{N}}$ are related by the same permutation matrix that has allowed the movement of the first row and column to the last, eigenvectors of $\widehat{\boldsymbol{N}'}$ and $\hat{\boldsymbol{N}}$ only differ by the arrangement of their elements. Given that the normalized eigenvectors of $\hat{\boldsymbol{N}}$ are $\boldsymbol{\xi}_\alpha = \left(A_{\alpha x}, A_{\alpha y'}, A_{\alpha z'}, \phi_\alpha, L_{\alpha x}, L_{\alpha y'}, L_{\alpha z'}, D_\alpha\right)^T$, eigenvectors of the permutated $\widehat{\boldsymbol{N}'}$ now have their elements arranged as $\boldsymbol{\xi}'_\alpha = \left(A_{\alpha y'}, A_{\alpha z'}, \phi_\alpha, L_{\alpha x}, L_{\alpha y'}, L_{\alpha z'}, D_\alpha, A_{\alpha x}\right)^T$.

The eigenvalue problem of $\widehat{\boldsymbol{N}'}$ can be defined as,

$$\det\left(\widehat{\boldsymbol{N}'} - p_\alpha \hat{\boldsymbol{I}}_{\boldsymbol{8}}\right) = \det\left(\begin{bmatrix} -p_\alpha \hat{\boldsymbol{I}}_{\boldsymbol{4}} & \widehat{\boldsymbol{B}'} \\ \widehat{\boldsymbol{C}'} & -p_\alpha \hat{\boldsymbol{I}}_{\boldsymbol{4}} \end{bmatrix}\right) = 0. \tag{S13}$$

Using Schur's complement, Equation S13 is rewritten as,

$$\det\left(\widehat{\boldsymbol{N}'} - p_\alpha \hat{\boldsymbol{I}}_{\boldsymbol{8}}\right) = \det\left(-\hat{\boldsymbol{I}}_{\boldsymbol{4}}\right).\det\left(\widehat{\boldsymbol{C}'}\widehat{\boldsymbol{B}'} - {p_\alpha}^2 \hat{\boldsymbol{I}}_{\boldsymbol{4}}\right) = 0. \tag{S14}$$

Here, we have an eigenvalue equation

$$\widehat{\boldsymbol{C}'}\widehat{\boldsymbol{B}'}x = {p_\alpha}^2 x \tag{S15}$$

for the matrix product $\widehat{\boldsymbol{C}'}\widehat{\boldsymbol{B}'}$, with x the eigenvectors and ${p_\alpha}^2$ the eigenvalues. Multiplying $x^\dagger \widehat{\boldsymbol{B}'}$ from the left to Equation S15, and we obtain

$$x^\dagger \widehat{\boldsymbol{B}'}\widehat{\boldsymbol{C}'}\widehat{\boldsymbol{B}'}x = p_\alpha^2 x^\dagger \widehat{\boldsymbol{B}'}x,$$

which leads to $\frac{x^{\dagger}\widehat{\boldsymbol{B}'}\widehat{\boldsymbol{C}'}\widehat{\boldsymbol{B}'}x}{x^{\dagger}\widehat{\boldsymbol{B}'}x} = p_{\alpha}^{2}$.

Both $\widehat{\boldsymbol{B}'}$ and $\widehat{\boldsymbol{B}'}\widehat{\boldsymbol{C}'}\widehat{\boldsymbol{B}'}$ are real symmetric matrices, so are Hermitian. Consequently, the left hand side of the equation has to be real. So all four eigenvalues p_{α}^{2} have to be real in Equation S14, and each pair of p_{α} is either purely real $\pm|l|$ or purely imaginary $\pm i|l|$, where $l \in \mathbb{R}$.

Only purely imaginary eigenvalues of $\widehat{\boldsymbol{N}}$ would be of interest here, that would support the construction of surface acoustic waves. From a pair of purely imaginary eigenvalues p_{α}, one can recover $p_{\gamma+4} = -p_{\gamma} = p_{\gamma}^{*}$ of Equation S7. For $\widehat{\boldsymbol{N}'}$, we denote eigenvectors $\boldsymbol{\xi}'_{\gamma}$ and $\boldsymbol{\xi}'_{4+\gamma}$ as $\boldsymbol{\xi}'_{\gamma} = (\boldsymbol{P}, \boldsymbol{Q})$ and $\boldsymbol{\xi}'_{4+\gamma} = (\boldsymbol{P}^{*}, \boldsymbol{Q}^{*})$ with $\boldsymbol{P}$, $\boldsymbol{Q}$, $\boldsymbol{P}^{*}$, and $\boldsymbol{Q}^{*}$ each a four-component vector.

To understand how the eigenvectors for a given pair of imaginary eigenvalues are related, let us assume the eigenvector corresponding to $+i|l|$ be $(\boldsymbol{P}, \boldsymbol{Q})$, and $-i|l|$ be $(\boldsymbol{P}^{*}, \boldsymbol{Q}^{*})$. Then,

$$\begin{bmatrix} \widehat{\boldsymbol{0}} & \widehat{\boldsymbol{B}'} \\ \widehat{\boldsymbol{C}'} & \widehat{\boldsymbol{0}} \end{bmatrix} \begin{bmatrix} \boldsymbol{P} \\ \boldsymbol{Q} \end{bmatrix} = i|l| \begin{bmatrix} \boldsymbol{P} \\ \boldsymbol{Q} \end{bmatrix}$$
$$\Rightarrow \widehat{\boldsymbol{B}'}\boldsymbol{Q} = i|l|\boldsymbol{P}$$
$$\widehat{\boldsymbol{C}'}\boldsymbol{P} = i|l|\boldsymbol{Q}, \tag{S16}$$

and,

$$\begin{bmatrix} \widehat{\boldsymbol{0}} & \widehat{\boldsymbol{B}'} \\ \widehat{\boldsymbol{C}'} & \widehat{\boldsymbol{0}} \end{bmatrix} \begin{bmatrix} \boldsymbol{P}^{*} \\ \boldsymbol{Q}^{*} \end{bmatrix} = -i|l| \begin{bmatrix} \boldsymbol{P}^{*} \\ \boldsymbol{Q}^{*} \end{bmatrix}$$
$$\Rightarrow \widehat{\boldsymbol{B}'}\boldsymbol{Q}^{*} = -i|l|\boldsymbol{P}^{*},$$
$$\widehat{\boldsymbol{C}'}\boldsymbol{P}^{*} = -i|l|\boldsymbol{Q}^{*}. \tag{S17}$$

We now rewrite vectors $\boldsymbol{P}$ and $\boldsymbol{Q}$ as $\boldsymbol{P} = \widehat{\boldsymbol{P}_{\theta}}\boldsymbol{P}'$ and $\boldsymbol{Q} = \widehat{\boldsymbol{Q}_{\phi}}\boldsymbol{Q}'$, where $\boldsymbol{P}'$ and $\boldsymbol{Q}'$ are column vectors containing the modulus of each element in $\boldsymbol{P}$ and $\boldsymbol{Q}$ respectively, $\widehat{\boldsymbol{P}_{\theta}}$ and $\widehat{\boldsymbol{Q}_{\phi}}$ are diagonal matrices containing the complex phase of each element as shown bellow,

$\widehat{\boldsymbol{P}_{\theta}} = diag(e^{i\theta_1}, e^{i\theta_2}, e^{i\theta_3}, e^{i\theta_4})$, and $\widehat{\boldsymbol{Q}_{\phi}} = diag(e^{i\phi_1}, e^{i\phi_2}, e^{i\phi_3}, e^{i\phi_4})$.

The first part of Equation S16 can be rewritten as,

$$\widehat{\boldsymbol{B}'}\widehat{\boldsymbol{Q}_{\phi}}\boldsymbol{Q}' = i|l|\widehat{\boldsymbol{P}_{\theta}}\boldsymbol{P}'.$$

Multiplying $\widehat{\boldsymbol{P}_{\theta}}^{\dagger}$ from the left on both sides, we have

$$\widehat{\boldsymbol{P}_{\theta}}^{\dagger}\widehat{\boldsymbol{B}'}\widehat{\boldsymbol{Q}_{\phi}}\boldsymbol{Q}' = i|l|\widehat{\boldsymbol{P}_{\theta}}^{\dagger}\widehat{\boldsymbol{P}_{\theta}}\boldsymbol{P}' = i|l|\boldsymbol{P}'. \tag{S18}$$

Similarly, multiplying $\widehat{\boldsymbol{P}_{\theta}}$ from the left to Equation S17, we get

$$\widehat{\boldsymbol{P}_{\theta}}\,\widehat{\boldsymbol{B}'}\widehat{\boldsymbol{Q}_{\phi}}^{\dagger}\boldsymbol{Q}' = -i|l|\,\widehat{\boldsymbol{P}_{\theta}}\,\widehat{\boldsymbol{P}_{\theta}}^{\dagger}\boldsymbol{P}' = -i|l|\boldsymbol{P}', \tag{S19}$$

Equations S18 and S19 lead to $\widehat{\boldsymbol{P}_{\theta}}^{\dagger}\widehat{\boldsymbol{B}'}\widehat{\boldsymbol{Q}_{\phi}} = -\widehat{\boldsymbol{P}_{\theta}}\,\widehat{\boldsymbol{B}'}\widehat{\boldsymbol{Q}_{\phi}}^{\dagger}$.

Comparing the individual elements of the matrices on both sides, we have,

$$B'_{jk}e^{i(\phi_k-\theta_j)} = -B'_{jk}e^{-i(\phi_k-\theta_j)},$$

where, $B'_{jk}$is the element belonging to j-th row and k-th column of $\widehat{\boldsymbol{B}'}$. Then, $e^{i2(\phi_k-\theta_j)} = -1$, and we have,

$(\phi_k - \theta_j) = n\pi + \frac{\pi}{2}$, with n an integer. (S20a)

From Equation S20a, it can be derived that,

$$(\phi_k - \phi_j),\ (\theta_{k'} - \theta_{j'}) = m\pi, \text{ where } m \in \mathbb{Z}. \tag{S20b}$$

We have established that for elements of $\boldsymbol{P}$ (and $\boldsymbol{Q}$), the phases differ only by integer multiples of π, and the phase difference between two elements of vectors $\boldsymbol{P}$ and $\boldsymbol{Q}$ respectively must be $n\pi + \frac{\pi}{2}$. The consequence of this phase relationship will be discussed in Section 4 together with the case in Section 3.

3. Scenarios of non-reciprocal SAW states

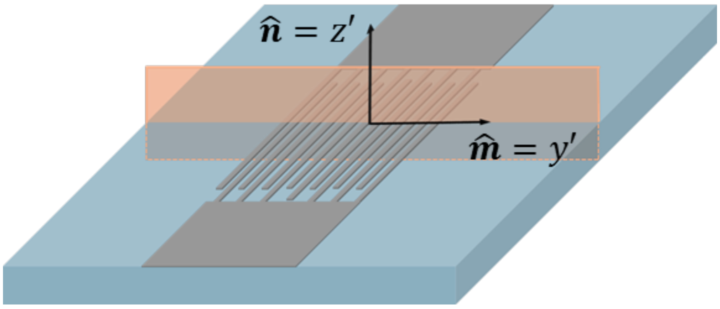

Figure S2: A schematic of non-reciprocal SAW propagation. In the specific case here, both $\hat{\boldsymbol{m}}$ and $\hat{\boldsymbol{n}}$ are confined to the mirror plane (orange) and are oriented along orthogonal axes y' and z'.

Now we survey the scenarios in which neither $\hat{\boldsymbol{m}}$ nor $\hat{\boldsymbol{n}}$ vectors are parallel to the crystalline x-axis (Fig. S2). As special cases, we examine where the x-axis is parallel to $\hat{\boldsymbol{m}} \times \hat{\boldsymbol{n}}$. Under this configuration, $\hat{\boldsymbol{m}}$ and $\hat{\boldsymbol{n}}$ are both within the y-z mirror plane and could be rotated by an in-plane angle. So we can define $\hat{\boldsymbol{m}} = y'$ and $\hat{\boldsymbol{n}} = z'$ and use the (x, y', z') coordinate system for our $\widehat{\boldsymbol{N}}$ matrix indices, which takes the form,

$$\widehat{\boldsymbol{N}} = \begin{bmatrix} \times & 0 & 0 & 0 & \times & 0 & 0 & 0 \\ 0 & 0 & \times & \times & 0 & \times & \times & \times \\ 0 & 1 & \times & \times & 0 & \times & \times & \times \\ 0 & 0 & \times & \times & 0 & \times & \times & \times \\ \times & 0 & 0 & 0 & \times & 0 & 0 & 0 \\ 0 & \times & \times & \times & 0 & 0 & 1 & 0 \\ 0 & \times & \times & \times & 0 & \times & \times & \times \\ 0 & \times & \times & \times & 0 & \times & \times & \times \end{bmatrix}. \tag{S21}$$

For Equation S21, we again can make a permuation transformation to exchange the second and fifth columns (and rows). This transforms $\widehat{\boldsymbol{N}}$ into a block matrix form,

$$\widehat{\boldsymbol{N}'} = \begin{bmatrix} \times & \times & 0 & 0 & 0 & 0 & 0 & 0 \\ \times & \times & 0 & 0 & 0 & 0 & 0 & 0 \\ 0 & 0 & 0 & \times & \times & \times & \times & \times \\ 0 & 0 & 1 & \times & \times & \times & \times & \times \\ 0 & 0 & 0 & \times & \times & \times & \times & \times \\ 0 & 0 & \times & \times & \times & 0 & 1 & 0 \\ 0 & 0 & \times & \times & \times & \times & \times & \times \\ 0 & 0 & \times & \times & \times & \times & \times & \times \end{bmatrix}. \tag{S22}$$

The eigenvectors are now formatted as $\boldsymbol{\xi}'_\alpha = \left(A_{\alpha x}, L_{\alpha x}, A_{\alpha y'}, A_{\alpha z'}, \phi_\alpha,\ L_{\alpha y'}, L_{\alpha z'}, D_\alpha\right)^T$, which implies that $A_{\alpha x}$ and $L_{\alpha x}$ are decoupled from other components and are only dependent on each other. With both $\hat{\boldsymbol{m}}$ and $\hat{\boldsymbol{n}}$ confined to the mirror plane, this represents a horizontal shear wave along $\hat{\boldsymbol{m}} \times \hat{\boldsymbol{n}}$, which is a purly elastic wave without a dependance on the piezolelectric tensor. SAW states constructed

from the rest of the components are completely confined to the mirror plane, which are the well-known piezoelectric Rayleigh waves.

The current $\widehat{\boldsymbol{N}}$ matrix form of Equation S22 cannot be further simplified as in Equations S12-S15. From Section 1, the eigenvalues thus are only complex conjugates to each other but not necessarily purely imaginary as in Equation S15. There is also no longer a phase relationship between components ($\boldsymbol{A}_{\alpha}, \phi_{\alpha}, \boldsymbol{L}_{\alpha}, D_{\alpha}$) of a given eigenvector similar to Equation S20.

For other geometries besides the scenarios in Sections 2 and 3, the SAW states will be the asymmetric type because of the projection from the current configuration of non-reciprocity.

4. Substrate geometry's influence on reflection coefficients

In this Section, we examine reflection coefficients of scenarios listed in Sections 2 and 3, which provide an understanding of the reciprocity (and non-reciprocity) observed in the main text. In Section 2, we observe that for the two scenarios of reciprocity, there is a strict phase relationship between elements of the single eigenvector that define the SAW for a substrate of $3m$ point group symmetry. These eigenvectors affect the SAW reflection coefficient $r^{(\pm)}$ on IDT, which are defined in Refs. [2, 19] as,

$$r^{(\pm)} = R_e^{(\pm)}(\beta) + R_m^{(\pm)}(\beta)\,\frac{h_{metal}}{\lambda_0},$$

$$\text{and } R_m^{(\pm)} = \frac{2\pi\sin(\pi\beta)}{\varepsilon_\infty}\frac{\Delta v}{v}\left\{\left[\rho v^2 + 4\mu\left(1-\frac{\mu}{\lambda+2\mu}\right)\right]{e_{\hat{m}}^{(\pm)}}^2 + [\rho v^2+\mu]{e_{\hat{m}\times\hat{n}}^{(\pm)}}^2 + \rho v^2 {e_{\hat{n}}^{(\pm)}}^2\right\}, \qquad \text{(S23)}$$

where $r^{(\pm)}$ is the total reflection, $R_e^{(\pm)}$ is the electrical component, $R_m^{(\pm)}$ is the mechanical component, β the metallization ratio, λ and μ are Lamé coefficients, ρ is the density of IDT material, h_{metal} is the thickness of the IDT, λ_0 is the wavelength of the SAW, and $e_i^{(\pm)}$ are the components of the vector $\boldsymbol{e}^{(\pm)} = \boldsymbol{A}^{(\pm)}/\phi^{(\pm)}$, where $i = \hat{m}, \hat{m}\times\hat{n}$ or $\hat{n}$.

The consequence of Equation S20 to Equation S23 is that components of $\boldsymbol{e}^{(\pm)}$ along two opposite directions must have the same magnitude, but also a phase difference of either $n\pi$ or $(n\pi + \pi/2)$. Then, the quantity $(\boldsymbol{e}^{(\pm)})^2$ would be purely real. According to Ref. [2], this implies that there cannot be any NSPUDT effect under these geometries. However, in the non-reciprocal case, the phase relation between $\boldsymbol{A}^{(\pm)}$ and $\phi^{(\pm)}$ is arbitrary, so it allows $(\boldsymbol{e}^{(\pm)})^2$ to be complex. Thus, these geometries allow for the amplification of non-reciprocity (asymmetry) between SAW states, based on multiple internal reflections of SAW within the same IDT. Our experimental results in the main text confirm that the asymmetry grows with both the height and the number of fingers of IDT, as they both promote internal reflections within the IDT.
